# Cross-sectional and longitudinal analysis of health-related quality of life (HRQoL) in senior and geriatric dogs

**DOI:** 10.1371/journal.pone.0301181

**Published:** 2024-09-04

**Authors:** Alejandra Mondino, Chin-Chieh Yang, Katherine E. Simon, Gilad Fefer, James Robertson, Margaret E. Gruen, Natasha J. Olby

**Affiliations:** 1 Department of Clinical Sciences, College of Veterinary Medicine, North Carolina State University, Raleigh, North Carolina, United States of America; 2 Biostatistics Administrative Department, College of Veterinary Medicine, North Carolina State University, Raleigh, North Carolina, United States of America; Texas A&M University College Station, UNITED STATES OF AMERICA

## Abstract

Advancements in veterinary medicine have resulted in increased life spans for dogs, necessitating a better understanding of quality of life for older dogs. This study aimed to evaluate quality of life (QoL) progression and its potential association with mortality in senior and geriatric dogs. The Canine Owner-Reported Quality of Life Questionnaire (CORQ), consisting of 17 questions across four domains (vitality, companionship, pain, and mobility) was employed. Higher scores indicated better quality of life, with 7 as the highest potential score for each question. In a cross-sectional analysis including 92 dogs, we found an inverse correlation between overall CORQ (and all domain scores) and fractional lifespan. The domain of vitality demonstrated the lowest scores, while companionship exhibited the highest. A longitudinal analysis, including 34 dogs, revealed that when dogs reach the geriatric stage (100% of their calculated lifespan), their expected overall CORQ is 5.95 out of 7, and dogs are expected to have a monthly decline of 0.05 units in the score. Cox proportional hazard analysis demonstrated a significant association between overall CORQ scores and mortality, with dogs scoring below 5.35 being at a higher risk of mortality. This study underscores the association between aging, declining quality of life, and increased mortality risk in aging dogs.

## Introduction

With advancements in veterinary medicine and an increased focus on preventive care, the lifespan of pet dogs is increasing. A recent study found that the median lifespan in pet dogs is 15.4 years [1], while older studies found a mean lifespan of 8.5 to 12.5 years [2–4]. As dogs age, they may experience a decline in overall health and well-being. In fact, age is one of the most influential factors for developing degenerative diseases such as osteoarthritis (that can result in chronic pain and limited mobility) [5] and canine cognitive dysfunction [6, 7]. Additionally, dogs may suffer from sensory impairment such as reduced vision [8] and hearing [9]. Age-related diseases can significantly impact their quality of life [10, 11].

Several definitions of quality of life for humans have been proposed, and it is currently accepted that quality of life is a multilevel, dynamic, and complex concept. One of the definitions states it as “an overall general well-being that comprises objective descriptors and subjective evaluations of physical, material, social, and emotional well-being together with the extent of personal development and purposeful activity, all weighted by a personal set of values” [12]. However, this definition is difficult to apply from a veterinary perspective, and a narrower concept, the health-related quality of life (HRQoL), might be more suitable to evaluate in veterinary patients. HRQoL can be defined as the reflection of the impact of disease and treatment on disability, daily functioning, and the ability to live a fulfilling life [13, 14]. Several owner-completed HRQoL questionnaires have been developed and validated for dogs, most of them for specific pathological conditions such as cancer [15, 16], chronic pain [17], or atopic dermatitis [18], but also general questionnaires that do not focus on specific diseases [11, 19].

Little is known about HRQoL in older dogs, yet understanding it in this population, particularly its progression and associated factors, is crucial for making decisions about end-of-life care, including when to consider euthanasia. A recent study using the VetMetrica HRQoL questionnaire 19has shown that older dogs show an age-related decline in HRQoL [20]. In a previous study, our group has also demonstrated that HRQoL in older dogs was negatively correlated with age and that some domains of quality of life such as vitality and companionship are worse in dogs with hearing loss [9]. Nevertheless, these were cross-sectional studies and neither the progression of HRQoL over time nor its association with death in older dogs was evaluated. The aims of this study were to evaluate HRQoL in senior and geriatric dogs, to describe changes in HRQoL over time, and to determine its predictive value for all-cause mortality.

## Material and methods

### Study population

Dogs in this study were part of the longitudinal study of neuro-aging at the North Carolina State University (NCSU), College of Veterinary Medicine. This is an on-going study that assesses the effect of aging in different domains such as cognition, sensory impairments, mobility, activity patterns, and sleep. In this study dogs are evaluated at the NCSU every 6 months, as has been described in our previous research [21, 22]. The study was divided into two different phases. First, we performed a cross-sectional evaluation of HRQoL in older dogs and we examined the association between a 17-item questionnaire and a single-item numerical rating scale. We also determined the correlation between HRQoL scores and age. Second, we followed up dogs longitudinally and asked owners to complete the HRQoL questionnaire every approximately 6 months until the dogs died, or owners withdrew their dogs from the study. Owners were asked to report if their dog died, the date and the cause of death. From January 2019 to January 2023, companion dogs were recruited for both phases of the study at North Carolina State University (NCSU) College of Veterinary Medicine. To be recruited, dogs had to have a fractional lifespan of ≥ 0.75, meaning they were older than 75% of their expected lifespan. The expected lifespan of each dog was calculated using the formula proposed by Greer et al. (2007) [2], which takes into account their height and weight. The fractional lifespan of a dog was determined by dividing their chronological age by their expected lifespan and multiplying it by 100.

Exclusion criteria at the time of recruitment were history of medical conditions or presence of abnormalities likely to result in death in the next 6 months based on clinical evaluation and complete hematology and chemistry results. Dogs with stable chronic diseases such as chronic kidney disease, myxomatous mitral valve disease, osteoarthritis, were included in the study. Additionally, dogs exhibiting significant anxiety and aggression at the hospital, to the extent that they hindered the ability to conduct cognitive testing or physical examinations, were excluded. Owners of eligible dogs were asked to provide written informed consent after receiving a detailed explanation of the study. They were given the opportunity to ask any question they may have. The protocols were reviewed and approved by the NCSU Institutional Animal Care and Use Committee (IACUC # 18-109-O; 21-303-O and 21-376-O), and all protocols were adhered to during the study. Humans were not used in this research because all data collected pertained to dogs, not humans. This work was categorized as “Not Human Subject Research”.

### Health-related quality of life questionnaire

For this study we used a HRQoL questionnaire [16], the Canine Owner-Reported Quality of Life Questionnaire (CORQ) already used by our group [9]. As in our previous study one question was modified by replacing the word "treatment" with "anxiety”. This was done because the CORQ was developed originally for dogs with cancer, and we did not study this population. All the remaining questions were maintained as in the original version of the questionnaire. The questionnaire is composed of four domains, including vitality, companionship, pain, and mobility, each containing a variable number of questions, for a total of 17 questions (Table 1). The score for each question corresponds to the number of days in the past week that the owner noticed a specific behavior described in the question. Every question was, therefore, scored from 0 to 7. As some of the questions reflected behaviors that could indicate either better or worse HRQoL, all items were scored such that higher scores were associated with a better response (i.e. higher score reflected a better HRQoL). For instance, the score for a negative behavior (like decreased appetite) was calculated by subtracting the owner’s response from 7. The overall CORQ and the score of each domain was calculated by averaging the scores as shown in Table 1.

**Table 1:** Canine Owner-Reported Quality of Life Questionnaire (CORQ) questions for each individual domain and the score calculation.

Domain A: Vitality = [A1 + A2 + A3 + (7-A4) + (7-A5)] / 5
A1. My dog did his/her favorite activities
A2. My dog was playful
A3. My dog acted like his/her normal self
A4. Anxiety interfered with life*
A5. My dog had a lack of energy
Domain B: Companionship = [B1 + B2 + B3 + (7-B4) + (7-B5) + (7-B6)] / 6
B1. My dog enjoyed being near me
B2. My dog showed a normal amount of affection
B3. My dog enjoyed being pet or touched
B4. My dog’s appetite was decreased
B5. My dog was reluctant to get up
B6. My dog did not eat his/her normal food
Domain C: Pain = [C1 + (7-C2)] / 2
C1. My dog slept well at night
C2. My dog had pain or discomfort
Domain D: Mobility = [(7-D1) + (7-D2) + (7-D3) + (7-D4)] / 4
D1. My dog fell or lost his/her balance
D2. My dog had trouble going for a walk
D3. My dog had trouble getting up or lying down
D4. My dog had trouble getting comfortable
Overall CORQ = [A1 + A2 + A3 + (7-A4) + (7-A5) + B1 + B2 + B3 + (7-B4) + (7-B5) + (7-B6) + C1 + (7-C2) + (7-D1) + (7-D2) + (7-D3) + (7-D4)] / 17

* Note: Since the questionnaire was developed originally for dogs with cancer and this was not the population we were studying. The word "treatment" was replaced by "anxiety"

In alignment with a prior study [16], owners were asked to give a general impression of their dog’s quality of life over the past week, using a single-item numerical rating scale from 1 to 10, where a higher score indicates a better quality of life.

### Statistical analysis

For the cross-sectional analysis, we evaluated each dog’s initial visit. The raw data used in this analysis is provided in S1 Table. Firstly, to assess the feasibility of using a single-item numerical rating scale as a reliable alternative when time constraints prohibit the use of the full 17-question CORQ questionnaire, we examined the correlation between scores obtained from the overall CORQ and the numerical rating scale. Additionally, we calculated the correlation between each CORQ domain and the numerical rating scale to identify which domain owners prioritized more when assessing the overall quality of life of their dogs. Furthermore, we explored the correlation between the scores in the questionnaires and two age estimation methods (chronological age and fractional lifespan) to investigate how CORQ and the numerical rating scale scores change with aging. Spearman correlation was employed for these correlation analyses. To address the issue of multiple comparisons, a Bonferroni correction was applied.

In the longitudinal analysis (raw data provided in S2 Table), we used linear mixed models with random intercepts to examine the longitudinal changes in the overall CORQ, each CORQ domain, and the single-item numerical rating scale. The models incorporated the variables of baseline fractional lifespan and time. The random intercept was specified as the dog ID, allowing for individual variability in the baseline level of the outcome measures. The baseline fractional lifespan was centered at 100%, and time was scaled at the unit of month. Consequently, the coefficient of time represents the estimated change for every 1-month increment for a dog who has reached their expected lifespan, or, in other words, who has just become geriatric according to the AAHA guidelines [23].

Cox proportional hazards regression models were employed to investigate the relationship between the overall CORQ score and all-cause death. The model was adjusted for fractional lifespan to account for its potential influence. To identify an optimal cut point for the overall CORQ, we utilized the "survminer" R package and employed the "surv_cutpoint" command, which used maximally selected rank statistics [24, 25]. This function evaluates different cut points to differentiate two groups of dogs (at low and at high risk of dying) based on different values of the overall CORQ score. It chooses the cut point that provides the best separation of the two groups survival curves. Subsequently, an additional Cox proportional hazards analysis was conducted using these two categories as predictors of all-cause mortality. The raw data used in this article is provided in S1 and S2 Tables.

## Results

### Cross sectional analysis

#### Study population

The study included a total of 92 dogs, comprising 49 spayed female dogs, 41 castrated male dogs, 1 intact female dog, and 1 intact male dog. The chronological age of the dogs ranged from 9.6 to 16.4 years (mean = 12.84 ± 1.61), while the fractional lifespan ranged from 77% to 126% (mean = 104 ± 12%). The breeds represented in the study were as follows: 42 mixed breed dogs, 8 Labrador Retrievers, 7 Golden Retrievers, 5 Beagles, 3 Australian Shepherds, 3 Basset Hounds, 3 Border Collies, 2 American Staffordshire Terriers, 2 Australian Cattle dogs, 2 German Shepherds, 2 Jack Russell Terriers, and one of each of the following breeds: Bernese Mountain dog, Chow-chow, Collie, Dachshund, Foxhound, German Shorthair Pointer, Great Dane, Great Pyrenees, Irish Setter, Pomeranian, Brittany Spaniel, Rhodesian Ridgeback, and Siberian Husky.

**Fig 1:**
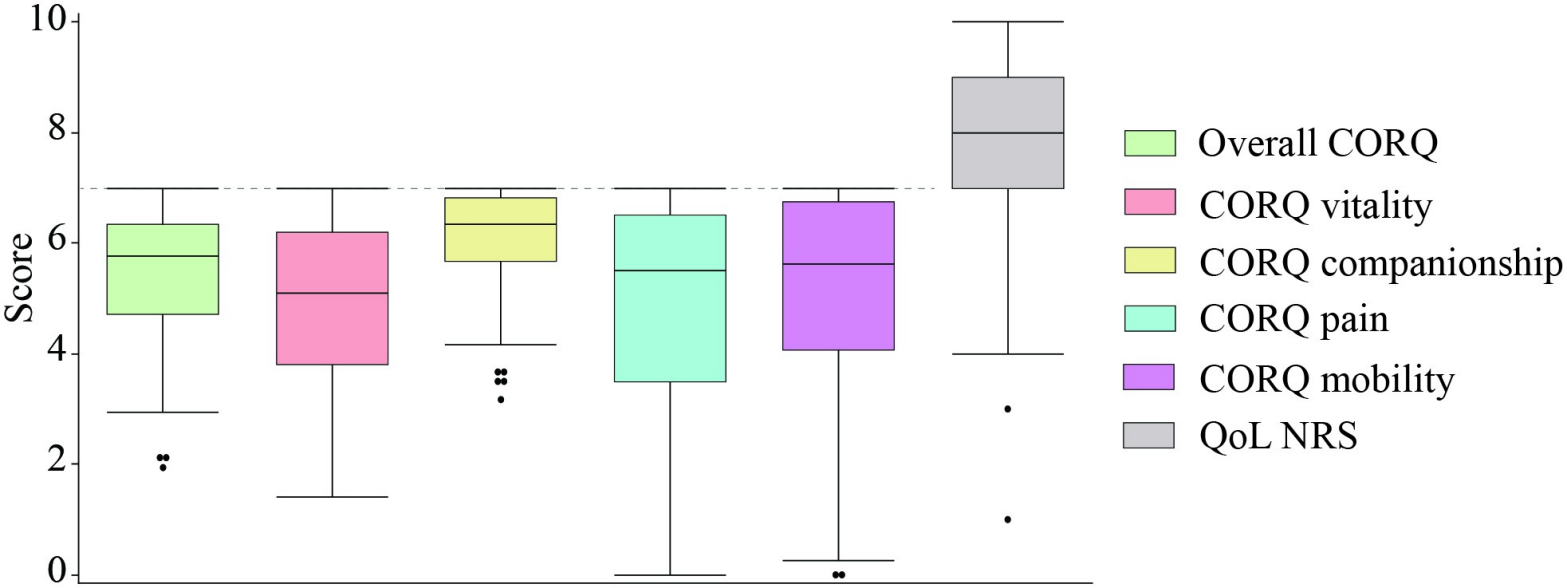
Box plot depicting the distribution of questionnaire scores in the 92 dogs included in the study. Note the difference in score range between CORQ and the quality of life (QoL) numerical rating scale (NRS). CORQ scores range from 0 to 7, and the numerical rating scale range from 0 to 10. The box represents the interquartile range, with the lower edge of the box indicating the 25^th^ percentile and the upper edge representing the 75^th^ percentile. The horizontal line within the box represents the median score. Whiskers extend from the box to indicate the range of the data, excluding any outliers. Outliers are represented as individual dots outside the whiskers. Note the upper limit of the CORQ scale is denoted by a dashed line.

The distribution of the questionnaire scores was found to be non-normal. Fig 1 presents the overall CORQ and each CORQ domain score as well as the numerical rating scale. The overall CORQ median score was 5.76 (range: 1.94–7). The domain of vitality exhibited lowest score (median 5.1, range 1.4–7) whereas the domain of companionship displayed highest scores (6.33 range: 3.17–7). The median score for CORQ pain was 5.5 (range: 0–7) and for mobility 5.6 (range: 0–7). The greatest variability was observed in these last two domains, with some dogs scoring relatively low in these areas (indicating poorer QoL).

#### Correlation between CORQ scores and the single-item numerical rating scale

As shown in Table 2, there was a significant positive correlation between the single-item numerical rating scale and the overall CORQ, indicating a strong association. Additionally, each individual domain of the CORQ also exhibited a positive correlation with the single-item scale. The domain of vitality demonstrated a strong correlation with the scale, while the remaining domains showed moderate correlations [26]. The domains of companionship and pain displayed the lowest correlation coefficients among all the domains.

**Table 2:** Spearman correlation between the single-time numerical rating scale and the total CORQ as well as each of the CORQ domains.

	Overall CORQ	CORQ vitality	CORQ companionship	CORQ pain	CORQ mobility
Single-item numerical rating scale	ρ = 0.79 adj p <0.001*	ρ = 0.74 adj p <0.001*	ρ = 0.59 adj p <0.001*	ρ = 0.60 adj p <0.001*	ρ = 0.69 adj p <0.001*

p values were adjusted (adj p) by Bonferroni multiple comparisons correction. Asterisks indicate significant differences.

#### Effects of age on HRQoL

Overall CORQ as well as the domains of vitality, companionship and pain were negatively correlated with both chronological age and fractional lifespan (Table 3). For all of them, the correlations were higher with fractional lifespan than with chronological age. The domain of mobility was only correlated with fractional lifespan. The single-item numerical rating scale was also negatively correlated with chronological age and fractional lifespan, and similar to CORQ, the correlation was much stronger for fractional lifespan than for chronological age.

**Table 3:** Spearman correlation between the overall CORQ, each CORQ domain and the single-item numerical rating scale with chronological age and fractional lifespan.

	Overall CORQ	CORQ vitality	CORQ companionship	CORQ pain	CORQ mobility	Single-item numerical rating scale
Chronological age	ρ = -0.42 adj p < 0.001*	ρ = -0.44 adj p < 0.001*	ρ = -0.33 adj p = 0.014*	ρ = -0.30 adj p = 0.046*	ρ = -0.28 adj p = 0.068	ρ = -0.41 adj p < 0.001*
Fractional lifespan	ρ = -0.61 adj p < 0.001*	ρ = -0.52 adj p < 0.001*	ρ = -0.39 adj p = 0.001*	ρ = -0.49 adj p < 0.001*	ρ = -0.52 adj p < 0.001*	ρ = -0.49 adj p < 0.001*

p values were adjusted by Bonferroni multiple comparisons correction. Asterisks indicate significant differences.

### Longitudinal analysis

Thirty-six of the 92 total dogs participated in the longitudinal study, but 2 were withdrawn from the study by their owners because of personal circumstances that rendered them unable to comply with the visit schedule. Among the remaining 34 dogs included in the analysis, 21 were spayed females and 13 were castrated males. Thirteen were mixed breed dogs, 3 were Labrador retrievers, there were 2 dogs of the following breeds: American Staffordshire terriers, border collies, golden retrievers and Jack russell terriers, and there was one of each of the following breeds: Australian cattle dog, Australian shepherd, basset hound, beagle, Brittany spaniel, dachshund, German shepherd, German shorthair pointer, Irish setter, pomeranian and Siberian husky.

**Fig 2:**
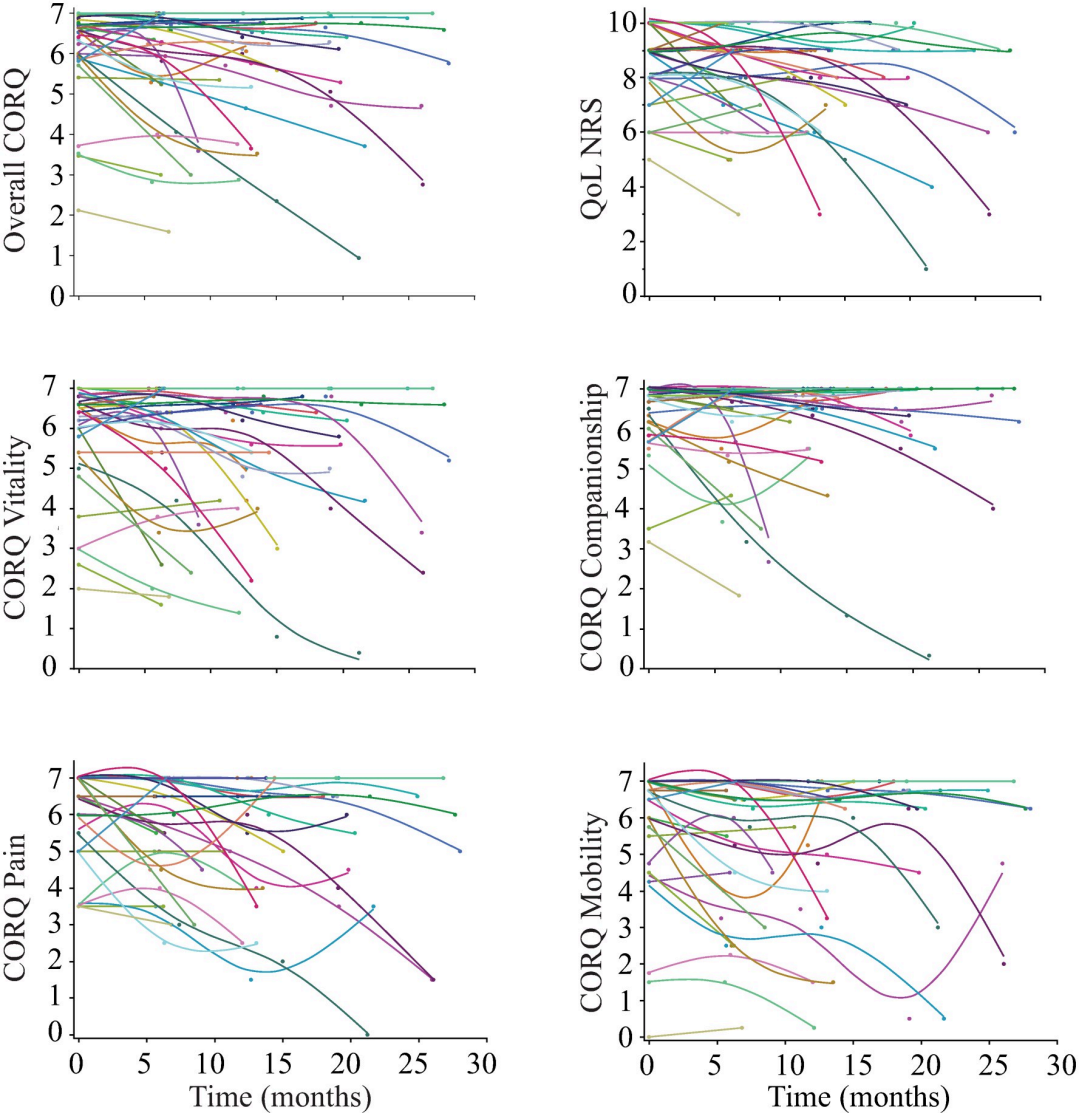
Individual’s trajectory of overall CORQ score in each domain and the single-item numerical rating scale (NRS).

The average age of these dogs at the study entry was 12.50 ± 1.66 years and their average fractional lifespan was 99 ± 12%. All of the dogs completed a minimum of two visits, 24 completed three visits, 15 completed 4 visits and 6 completed 5 visits. The average time between the first and the second visit was 6.42 ± 1.01 months, between the first and the third visit was 12.76 ± 1.30 months, between the first and the fourth visit was 19.0 ± 2.17 months and between the first and the fifth visit was 26.6 ± 1.16 months. Fourteen of the 36 dogs died during the study. On average, the time from the first visit to death was 12.49 ± 6.20 months. All the dogs who died were euthanized, 6 due to owner’s concerns about their dog’s quality of life, 5 due to cancer (1 hemangiosarcoma, 1 soft tissue carcinoma, 1 oral carcinoma, 1 mast cell tumor and 1 thoracic mass), 1 due to respiratory distress caused by chronic bronchitis, 1 due to congestive heart failure and 1 due to renal failure. The median CORQ overall at the visit before being euthanized was 3.70 (IQR: 2.97–5.30). The median vitality score was 3.8 (IQR: 2.05–5.45), the companionship score was 5.5 (IQR: 4.24–6.54), the pain score was 4.0 (IQR: 2.50–5.0) and, the mobility score was 3.62 (IQR: 1.87–4.81). The single-item numerical rating scale was 6.0 (IQR: 4.5–8). Fig 2 shows the trajectory of the overall CORQ score and each of the CORQ domains as well as the single-item numerical rating scale. As can be observed, most of the dogs experienced a reduction in their scores over time, with only a few showing an improvement in some of the scores at any specific visit.

Table 4 displays the mean decrease observed in the overall CORQ score, as well as in each CORQ domain and in the numerical rating scale score, for each subsequent visit in comparison to the initial visit. The domain of pain had the highest reduction in score over time.

**Table 4:** Reduction in scores relative to the initial visit. Data is presented as mean ± standard deviation.

	2^nd^ visit (n = 34)	3^rd^ visit (n = 24)	4^th^ visit (n = 15)	5^th^ visit (n = 6)
Overall CORQ	0.55 ± 1.26	0.81 ± 1.03	0.93 ± 1.35	1.49 ± 0.61
CORQ vitality	0.35 ± 0.93	0.93 ± 1.41	1.07 ± 1.46	1.46 ± 1.95
CORQ companionship	0.26 ± 0.91	0.51 ± 1.37	0.71 ± 1.63	0.52 ± 1.21
CORQ pain	0.57 ± 1.56	1.02 ± 1.25	1.03 ± 1.57	2.08 ± 2.37
CORQ mobility	0.57 ± 1.08	1.00 ± 1.68	1.05 ± 1.54	0.92 ± 1.56
Single-item numerical rating scale	0.41 ± 1.13	1.17 ± 1.68	1.53 ± 2.13	1.08 ± 1.49

The results of the regression analyses examining the role of time and fractional lifespan at baseline on HRQoL are presented in Table 5. When analyzing the overall CORQ we found that both time and baseline fractional lifespan had a significant negative association with the questionnaire score. Similar patterns were observed for the domains of vitality, companionship, and mobility. According to these results, dogs decline by an average of 0.05 points in overall CORQ score every month and by an average of 0.80 units for every 10% increase on their fractional lifespan at baseline.

**Table 5:** Results of the regression analysis examining the relationship between time and fractional lifespan at baseline and questionnaire scores.

Questionnaire	Variable	Estimate	SE	t value	Adj p value
Overall CORQ	Intercept	5.95	0.17	35.02	<0.001*
	Time (months)	-0.05	0.01	-5.82	<0.001*
	Baseline frac. Lifespan	-0.08	0.01	-6.31	<0.001*
CORQ vitality	Intercept	5.77	0.23	25.38	<0.001*
	Time (months)	-0.07	0.12	-5.52	<0.001*
	Baseline frac. Lifespan	-0.08	0.02	-4.76	<0.001*
CORQ companionship	Intercept	6.32	0.17	36.38	<0.001*
	Time (months)	-0.03	0.01	-3.27	0.027*
	Baseline frac. Lifespan	-0.06	0.01	-4.76	<0.001*
CORQ pain	Intercept	5.92	0.19	30.51	<0.001*
	Time (months)	-0.07	0.01	-5.87	<0.001*
	Baseline frac. Lifespan	-0.08	0.01	-5.72	<0.001*
CORQ mobility	Intercept	5.63	0.24	23.12	<0.001*
	Time (months)	-0.05	0.012	-4.37	<0.001*
	Baseline frac. Lifespan	-0.11	0.02	-5.88	<0.001*
Single-item numerical rating scale	Intercept	8.53	0.21	39.6	<0.001*
	Time (months)	-0.09	0.01	-5.76	<0.001*
	Baseline frac. Lifespan	-0.092	0.015	-5.94	<0.001*

SE: Standard error. P values were adjusted by Bonferroni multiple comparisons correction. Asterisks indicate significant differences.

**Fig 3:**
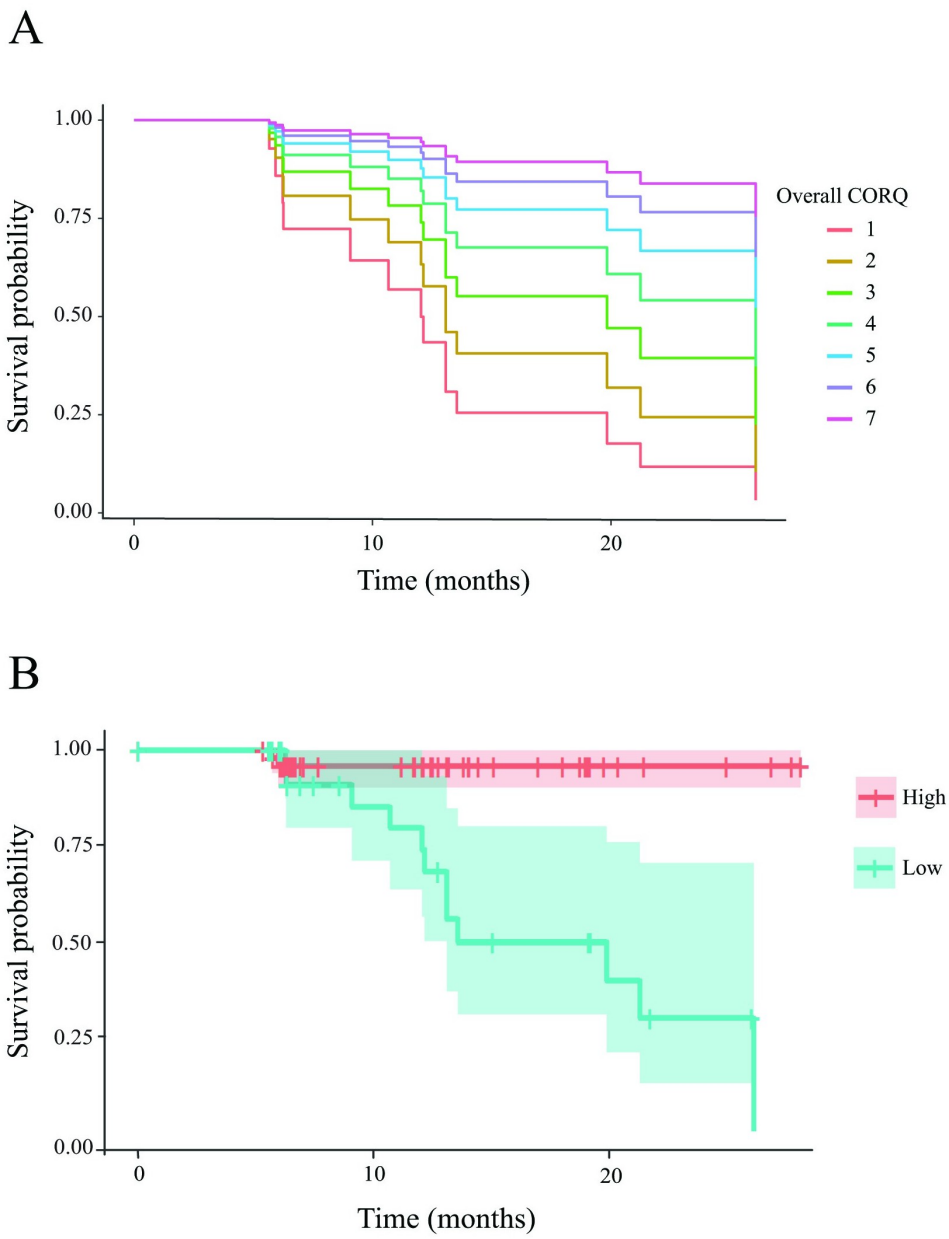
Survival plot for every score on the overall CORQ (A) and for the groups of dogs categorized as low overall CORQ and high overall CORQ based on the determined cut off value of 5.35 (B).

A Cox proportional hazard analysis was conducted on the overall CORQ score, with fractional lifespan considered as a confounding variable. We used the overall score because it encompasses all potential domains that may impact dogs’ HRQoL. This analysis showed that the hazard ratio (HR) for the overall CORQ was 0.56 (95% CI: 0.34–0.93, p = 0.023), indicating that for each one-unit decrease (worsening) in the score, the chances of dying increase by 79%. Conversely, the hazard ratio for fractional lifespan was 1.02 (95% CI: 0.93–1.10, p = 0.717), indicating that when CORQ scores are included in the model, there is no significant association between fractional lifespan and the risk of all-cause death. The model had a good fit (concordance index = 0.842) and was statistically significant (likelihood ratio test, p < 0.001). Fig 3A illustrates the survival curve for every single increment in the overall CORQ value.

By using the “surv_cutpoint” command in “survminer” R package, an overall CORQ cut off was determined. An overall CORQ score of 5.35 was found to be the optimal cut off for differentiating between high and low-risk groups for mortality. The dogs classified as having low total CORQ scores had a HR 15.44 times higher than the group with high total CORQ scores (95% CI: 2.31–102.99, p = 0.004). There was no significant effect of fractional lifespan, (HR = 1.01; 95% CI: 0.93–1.08, p = 0.906). This model also had a good fit (concordance index = 0.834) and was statistically significant (likelihood ratio test, p < 0.001). Fig 3B shows the survival curve for high and low CORQ categories.

## Discussion

In this study, our aim was to assess the HRQoL in senior and geriatric dogs. To accomplish this, we used a slightly modified version of the CORQ questionnaire and a single-item numerical rating scale that queries overall quality of life. We investigated the correlation between age and HRQoL, tracked the changes in HRQoL over time, and explored the impact of HRQoL on mortality. Our cross-sectional analysis revealed a significant correlation between single-item numerical rating scale and all domains of CORQ (p < 0.001), with vitality showing the strongest correlation (ρ = 0.74). All domains and overall CORQ were negatively correlated to fractional lifespan. The correlation with chronological age was weaker than with fractional lifespan for overall CORQ and all the domains, and it did not reach statistical significance for the domain of mobility. In the longitudinal analysis, all CORQ data were negatively correlated with time. The overall CORQ declined by 0.05 points every month and by 0.80 units for every 10% increase in their fractional lifespan. We also found the overall CORQ below 5.35 indicated a higher risk of all-cause death (HR = 15.44).

The original CORQ questionnaire was developed for use in cancer patients, which was not the population of dogs we focused on in our study. However, most of the questions in the original CORQ were generic and not exclusively related to the disease, except for a specific question within the vitality domain. This question asked about how much treatment interfered with dogs’ lives. To ensure that the questionnaire was more relevant to our study population, we substituted the term “treatment" with “anxiety”. This modification was made because research has shown that anxiety is one of the main behavioral concerns in older dogs [27] and it is one of the characteristics of canine cognitive dysfunction syndrome [28], which is highly prevalent in this age group [29]. By making this adjustment, we aimed to align the questionnaire more closely with the specific concerns of the dogs in our study without changing the overall interpretation of the questionnaire. The dogs in this study were part of a longitudinal study of neuro-aging that involved physical examinations, cognitive, sensory and mobility assessments and, therefore, those exhibiting higher levels of anxiety and aggression at time of initial evaluation for study inclusion that impede handling were excluded. Consequently, our findings might not represent the impact of severe anxiety on the quality of life of older dogs although it is important to note that if they developed anxiety during the course of the study, they were not removed from the study. We found that senior and geriatric dogs who did not suffer from any life-threatening disease at the time of enrollment in the study had a median overall CORQ of 5.76 out of 7. Similar to our findings in a previous study [9], dogs tended to score higher in the companionship domain than in the other domains, with no dog scoring less than 3 in this domain. The domain of companionship holds particular importance for dog owners: owners value engaging in shared activities with their dogs and experiencing reciprocal displays of affection. Many owners consider these interactions to be crucial for their dog’s overall wellbeing [30]. Additionally, a previous study involving dogs with spinal cord injury found that dog owners prioritize companionship over physical parameters when assessing their dogs’ quality of life [31]. In our population, the highest variability in scores was found in the domains of pain and mobility. Only for these 2 domains were there dogs who scored both the highest and the lowest possible score (i.e., score 7 and 0). This supports the importance of recognizing that pain or mobility impairment are not traits of normal aging and that they only occur in a subset of older dogs [32].

We also examined the relationship between the scores obtained from the CORQ questionnaire, which consisted of 17 questions across four different domains, and the ratings from the numerical rating scale that assessed the overall HRQoL in dogs. Our findings revealed a robust correlation between the overall CORQ questionnaire score and the numerical rating scale. This result agrees with the findings of Giuffrida et al. (2018) [16] showing that the overall CORQ correlated strongly with an owner-reported visual analog scale score of quality of life. This suggests that the numerical rating scale can serve as a convenient alternative for evaluating HRQoL in older dogs in situations where administering the whole questionnaire is not feasible. Studies in humans have also shown that single-item quality of life scales have good validity and reliability, however, multi-item questionnaires can generate more reliable responses over time and are more responsive to specific treatment effects than single-item scales [33, 34]. In veterinary medicine, using a single-item measure does not allow for evaluation of each domain of HRQoL, potentially resulting in the loss of valuable information. When evaluating individual domains of the CORQ questionnaire, we found that the domain of vitality, followed by mobility, exhibited the highest correlation with the single-item measure. This suggests that owners may prioritize aspects related to vitality and mobility in their overall assessments. In contrast, the domains of companionship and pain demonstrated the lowest correlation. The limited variability observed in the companionship domain among this population of dogs might explain why owners did not prioritize this aspect when evaluating their dogs’ quality of life. Additionally, owners may attribute relatively less importance to the role of pain in their overall assessment of their dogs’ quality of life, or they might not link mobility impairments with pain. A recent study discovered that dog owners are more adept at recognizing pain-related behaviors in young dogs compared to senior dogs. They struggle to associate behavioral changes in senior dogs with pain, potentially mistaking them for normal signs of aging, and maybe not realizing the impact of pain on the dogs’ quality of life [35]. However, given pain can also influence many of the responses to the questions in the other domains [36], the impact of pain is likely captured well by this questionnaire. Similarly, while one of the questions in the pain domain relates to sleep quality and sleep disruption has been associated with pain [37, 38], and is one of the easiest signs of pain for owners to recognize in older dogs [35], it can also be influenced by other factors such as canine cognitive dysfunction [39]. Future studies could evaluate the interplay of pain and cognitive dysfunction more specifically. As anticipated, our study shows a decline in the quality of life as dogs age. In line with these findings, Chen et al. (2023) [20] conducted a study using the Vetmetrica questionnaire and reported similar results; older dogs exhibited lower HRQoL than younger dogs. We observed a stronger correlation between the quality of life and fractional lifespan (which takes into consideration the dogs’ body size) compared with chronological age across all domains. In fact, the domains of pain and mobility were not significantly associated with chronological age, but they were with fractional lifespan. Similarly, the aforementioned study indicated that larger breed dogs experience a more pronounced age-related decline in HRQoL than smaller breed dogs. Therefore, large breed dogs seem to have a more rapid decline in quality of life, especially in the mobility and pain domains. In this regard, larger breeds and higher body weight have been associated with higher risk of osteoarthritis [5, 40]. We found that the overall CORQ score of 5.35 served as the most effective cutoff for distinguishing dogs at a higher risk of mortality. We have also shown that HRQoL is a more important mortality risk factor than fractional lifespan. This supports the findings of Pegram et al. 2021 [41] who also showed that poor QoL was a significant risk factor associated with euthanasia. This finding can provide valuable guidance for veterinarians and owners when considering interventions to improve HRQoL in dogs. Moreover, it can assist in making decisions regarding euthanasia, which is a crucial consideration since approximately 90% of end-of-life situations in dogs involve euthanasia rather than unassisted death [41].

We showed a reduction in QoL over time in consequent visits. Time between visits was approximately 6 months, however some dogs had more or less time between those due to schedule limitations. In order to consider the specific effect of time, we also used a mixed model analysis to demonstrate the rate of decline in HRQoL. Our findings revealed that overall CORQ exhibited a monthly decline of 0.05 points and that the score from which each dog started was dependent on their fractional lifespan. To the best of our knowledge, this study represents the first exploration of longitudinal changes in HRQoL in senior and geriatric dogs. These findings have important implications for clinical practice. The American Animal Hospital Association recommends that senior and geriatric dogs receive routine healthcare visits every six months [42], and we consider that incorporating validated QoL questionnaires into these visits can be very helpful. However, it is crucial for clinicians to have information on the expected changes in questionnaire scores over time to properly interpret the results and develop a management plan for the dog.

## Limitations

Our study has some limitations. First, we analyzed a small sample size in the longitudinal analysis; increasing the number of cases and extending the follow-up period would likely enhance the robustness of the results. Secondly, the CORQ questionnaire, originally developed and validated for cancer patients, was adapted for our study by modifying one question to make it more relevant to our study population. However, this modified version was not re-validated. Moreover, while the CORQ questionnaire has been reported to reliably assist in assessing the quality of life in dogs, it relies on owner responses, introducing potential bias. Additionally, our study focused primarily on evaluating normal aging in dogs without life-threatening diseases at the time of enrollment (with an expected survival time of less than 6 months) and the trajectory of change in dogs suffering significant health concerns has not been investigated. We did include dogs with chronic stable diseases and elderly dogs are at high risk of developing new conditions. We did not attempt to assess the effect of disease progression and treatment modifications on the QoL assessments given the size of our longitudinal cohort. Some of the fluctuation, particularly in the mobility domain, likely resulted from treatment changes. Future longitudinal studies in a larger cohort of dogs could include these variables in the analysis. We also excluded dogs who displayed signs of aggression or anxiety at the appointment that prevented them from being handled by the experimenters. By doing so, we may have biased our sample towards dogs with a higher level of engagement in social interactions and we did not evaluate QoL in dogs with existing severe anxiety. This could potentially explain the high scores assigned by owners in the domain of companionship in our population of dogs. Finally, in the longitudinal study, the assessment was performed approximately every 6 months, however due to schedule difficulties some dogs had more or less time between visits.

In summary, our study revealed an inverse correlation between HRQoL in senior and geriatric dogs and their fractional lifespan by using an owner-based questionnaire. We demonstrated that if owners are asked about their dogs’ overall quality of life, they appear to base their response on aspects of vitality and mobility. While the pain domain was not given as much weight according to this questionnaire, the influence of pain is likely also being captured in the other domains. We followed changes in HRQoL over time and demonstrated that a decline in HRQoL was associated with an increased risk of mortality. This result emphasizes the importance of regular HRQoL assessment in aging dogs.

Future investigations focusing on interventions will contribute to a deeper understanding of strategies to enhance or reduce the rate of decline of HRQoL in this population.

## Supporting information

S1 Table Cross sectional data. This excel file includes all the data analyzed in the cross sectional analysis.
(XLSX)

S2 Table Longitudinal data. This excel file includes all the data analyzed in the longitudinal analysis.
(XLSX)
